# Extracellular microcystin prediction based on toxigenic *Microcystis* detection in a eutrophic lake

**DOI:** 10.1038/srep20886

**Published:** 2016-02-15

**Authors:** Xin Dong, Siyu Zeng, Fei Bai, Dan Li, Miao He

**Affiliations:** 1 Environmental Simulation and Pollution Control (ESPC) State Key Joint Laboratory, School of Environment, Tsinghua University, Beijing 100084, China

## Abstract

Existing models for predicting microcystin concentration in water body generally use chlorophyll or cyanobacteria concentration as input variables, although microcystins only originate from toxigenic strains of a few species. Moreover, the nonconcurrency between harmful algal growth and toxin release has yet to be quantified. Therefore, this study explored a new prediction method that considers these toxin production mechanisms for the eutrophic Yangcheng Lake, a large-scale drinking water source in China. The Lake was monitored weekly at six sampling sites from July to October in 2012, including the detection of toxigenic *Microcystis* (expressed as mcyA copy number) by qPCR. Compared with chlorophyll a, cyanobacteria, and total *Microcystis* abundance, toxigenic *Microcystis* concentration was more significant in predicting extracellular microcystin. Site-specific nonlinear regression models that link mcyA to microcystins were established. Parameters for toxin release delay (i.e., one or two weeks) were embedded in these models. Further analysis ascribed the different release timescale to NH_3_-N:TN and TN:TP ratios of approximately 0.015 and 9.2, respectively, which may decrease the delay in microcystin release. Model applications in determining mcyA monitoring frequency and its warning thresholds were discussed.

Cyanobacterial bloom is caused by the excessive growth of cyanobacteria in freshwaters and estuaries, which significantly affects the quality of original water environment and the survival of aquatic organisms^1^. This phenomenon has been recognized as a global problem in sustaining lake ecosystems and human health^2^. *Microcystis* is a major attributor to cyanobacterial bloom and a predominant source of microcystins (MCs) in water bodies^3^. MCs are cyclic heptapeptide secondary metabolites that are highly stable and toxic^4^,^5^. A high dose or a long-term exposure to MCs leads to potential serious health problems, such as liver cancer^6^, pneumonia, and gastroenteritis^7^. In general, most MCs originate from *Microcystis*, *Anabaena*, and *Planktothrix*^8^,^9^,^10^. Cells that are lysed or damaged release large amounts of toxins into the water^11^,^12^. Thus, estimation of extracellular MC concentration is a vital precondition to ensure human and environmental health.

Chlorophyll a (Chla) is typically used to forecast MC concentration on the basis of both field studies and laboratory tests. Izydorczyk *et al.*^13^ found a correlation between Chla and MC concentration in drinking water sources, which they regarded as a basis for toxin prediction. Similarly, Ziegmann *et al.*^14^ utilized fluorescence fingerprints that are associated with Chla concentration to depict the variation in MCs produced at different growth phases. Long *et al.*^15^ formulated a model that is dependent on the growth rate of N-limited *Microcystis aeruginosa* to predict cell quotas of MCs in a batch culture experiment. Cell quotas of MCs, protein, Chla, cell dry weight, and cell volume were measured over a range of growth rates of one toxic strain. The model was used to estimate the maximum and minimum toxin production on the basis of a theoretical growth ratio. Another hydrodynamic ecosystem-coupled model that integrates the toxin production affected by TP, TN, and some dominant species of cyanobacteria and toxin decay was established to simulate MC concentration in a lake ecosystem^16^. *Microcystis* cells and other biological and chemical parameters were chosen as input variables to describe algal composition and to estimate toxin production on the basis of the growth pattern and level of limiting factors, such as temperature, nutrients, and wind direction.

Various prediction models identified elements that affect cyanobacterial growth or MC production. These models can characterize the variation and level of MCs to a certain degree. However, the variables used for toxin prediction have not been directly linked to MC production. For example, Chla was used as the biomass to represent all of the algae related to photosynthesis. Several cyanobacterial species can produce MCs during cyanobacterial bloom^8^,^9^,^10^. Toxins originate from toxigenic strains rather than all strains of these species^17^. Hence, Chla is apparently not the most relevant attributor to MC production. As a result, even if the correlation coefficient between Chla and the toxins is significant, the uncertainty level is relatively high, and the application of Chla is limited to lakes with absolute dominant toxigenic species of cyanobacteria^18^,^19^. In view of MCs originating from toxigenic cyanobacteria^20^, the amount of toxigenic strains is theoretically a suitable parameter for forecasting the MC level. Real-time quantitative PCR (qPCR) based on mcy gene sequences of *Microcystis* is a highly sensitive method for quantifying toxigenic strains of *Microcystis*^21^,^22^. This approach provides a solid foundation to elucidate the relationship between toxigenic *Microcystis* abundance and MC concentration.

Furthermore, a temporal discrepancy from strain growth to toxin release possibly exists because most MCs are only released into the water body after the lysis of toxigenic *Microcystis* cells. The inputs and outputs in existing models for extracellular MC prediction are generally synchronized variables. In other words, toxin level cannot be predicted without input assumed by model users. Therefore, a new model that describes MC release delay may allow the prediction of future toxin concentration on the basis of the current quantity of toxigenic *Microcystis*. Such a model contributes to warning the risk posed by MCs.

Toxigenic *Microcystis*, MCs, and other water quality items in the Yangcheng Lake were monitored in 2012 for this study. A new model that considers toxin release delay was proposed for predicting MC concentration on the basis of the amount of toxigenic *Microcystis* measured through qPCR. Quantification of the release delay enabled the estimation of MC concentration one or two weeks earlier than previous methods. Furthermore, the potential reason for the variation in delay period was discussed.

## Results and Discussion

### Temporal profile of toxigenic *Microcystis* and MC release delay

**Figure 1:**
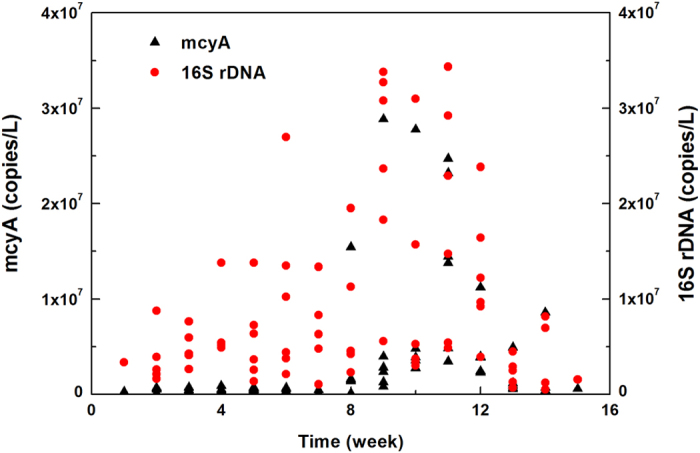
mcyA and 16S rDNA concentrations in the Yangcheng Lake during the sampling period. They both reached peaks on week 9 to 11 in September. For each week, data from 6 sites were shown.

During the sampling period, total *Microcystis* (expressed as 16S rDNA copy number) and toxigenic strains (expressed as mcyA copy number) in the Yangcheng Lake exhibited similar temporal trends; that is, they both showed peaks on week 9 to 11 in September (Fig. 1) with an average abundance of 1.5 × 10^7^ and 7.5 × 10^6^ copies/L, respectively. Total *Microcystis* grew gradually and steadily from week 4 to 11, while the toxigenic strains increased sharply after week 8. The proportion of toxigenic *Microcystis* to total strains reached approximately 69% at the peak compared with the 8% in other months, indicating that toxic genotypes achieved a higher growth in September. Because cell quotas for MCs vary with the growth rate of natural *Microcystis*^23^,^24^ and the toxicity effect increases by 10- to 1000-fold when the *Microcystis* community is changed to toxic strain predominance^25^,^26^, the monitoring results indicated that toxigenic strains were more likely turning into the exponential growth phase by the end of August and at the beginning of September, while intracellular toxins began to accumulate in this phase.

**Figure 2:**
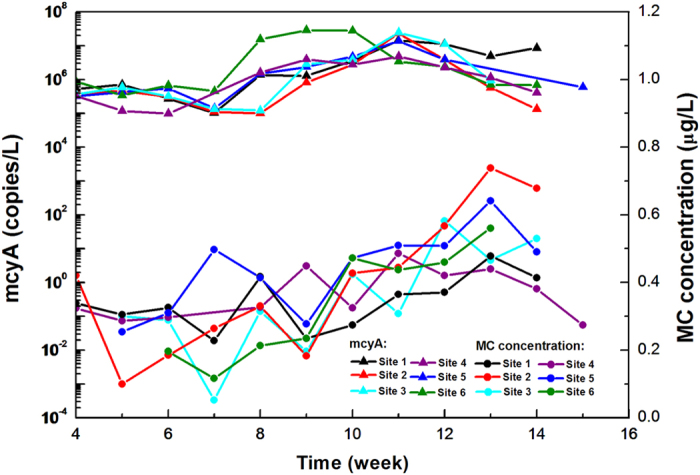
Change of mcyA (copies/L) and MC concentration (μg/L) in the Yangcheng Lake during the sampling period. MC concentrations reached the peaks one or two weeks after the mcyA’s maximum values showed.

Previous researchers found that the maximum extracellular MC concentration does not concurrently occur with peak value of mcyA copy number as well as of cyanobacterial abundance. Toxin concentration poorly correlates with these two variables (P > 0.05)^27^,^28^. In this work, the successive and long-term field monitoring study revealed the details of the inconsistency. Firstly, correlation analysis supported the previous findings. P-values of the hypothesis tests verified that neither the linear nor natural log-linear relationships between variables for abundance (including Chla, cyanobacteria, 16S rDNA, and mcyA) and MCs were significant (Table 1, see the column of *τ* = 0 for reference). Moreover, the monitoring data indicated a distinct time difference between the occurrence of the maximum values for cyanobacteria, 16S rDNA, mcyA, and MCs. Taking the mcyA as an example (Fig. 2), the mcyA and MC concentrations at the six sites gradually increased from week 7, and then the mcyA of all sites peaked around week 11. Afterward, MC concentrations steadily increased and reached the maximum values one or two weeks later, i.e. around week 12 or week 13, whereas mcyA copy numbers decreased.

In general, intracellular toxins increase with the increase in toxigenic *Microcystis* biomass and reach the maximal value at the end of the exponential growth phase^29^. By contrast, intracellular toxins are not immediately released. Most MCs are completely released into the water body when the mature cells of toxigenic *Microcystis* are injured to lyse or grow to death. For the nonconcurrency between toxigenic *Microcystis* growth and MC release was verified in the Yangcheng Lake, the extracellular MC concentration could only be well estimated through toxigenic *Microcystis* abundance by considering delay period.

### MC prediction models

In the current management framework, chlorophyll concentration or cyanobacteria abundance is generally used to predict MC concentration in the water. Although chlorophyll concentration and cyanobacteria are easily monitored, model performance requires careful validation, and high uncertainty impedes further application. To identify the best predictor for MC concentration estimation for the Yangcheng Lake, regression models were tested with different independent variables (Chla, cyanobacteria, 16S rDNA, and mcyA concentration) as input and MC concentration as output. Release delay was added as another parameter into each model to quantify the nonconcurrent phenomenon discussed in the previous section. The general model structure was described using the following equation:


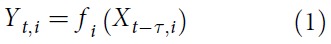


where *t* is the sampling time (week), *τ* is the delay period of MC release (week), *i* is the site number, *Y*_*t, i*_ is the concentration of MC (μg/L) at week *t* of site *i*, and *X*_*t−τ, i*_ is Chla (mg/L)/cyanobacteria (cells/L)/16S rDNA (copies/L)/mcyA (copies/L) at week (*t*−*τ*) of site *i*. Monitoring data were recorded weekly; thus, different delay periods (1 to 4 weeks, i.e., *τ* = 1, 2, 3, 4 weeks) for each sampling site were tested for the best data fitting.

**Figure 3:**
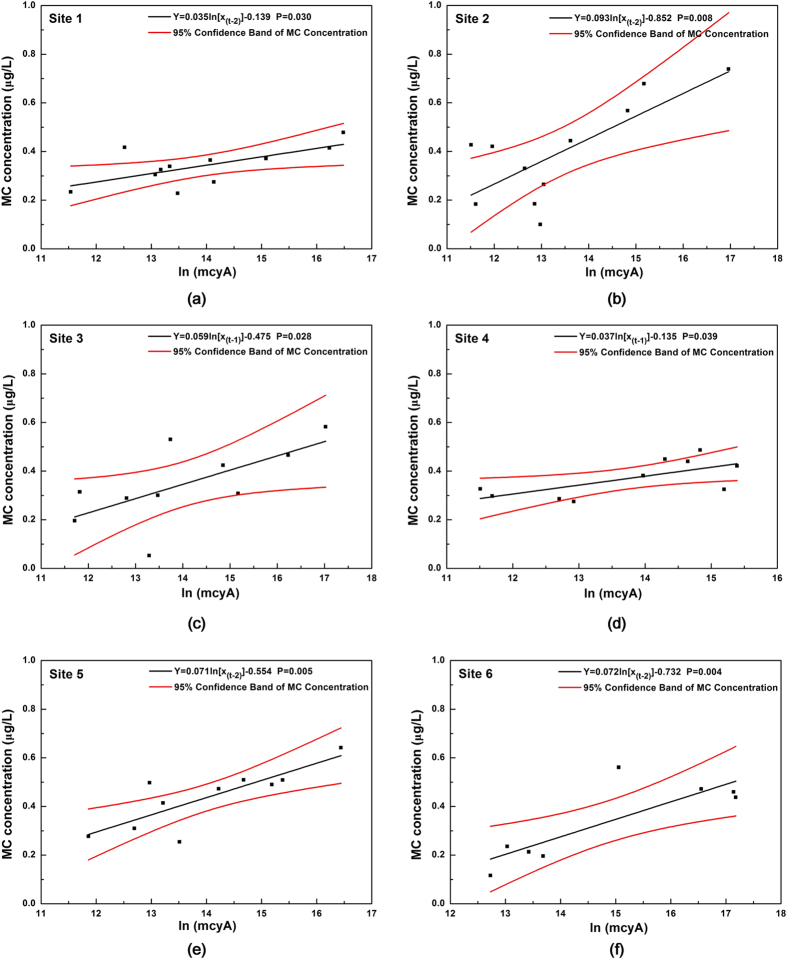
Regression models describing relationships between mcyA (copies/L) and MC concentration (μg/L) for (**a**) Site 1, (**b**) Site 2, (**c**) Site 3, (**d**) Site 4, (**e**) Site 5, and (**f**) Site 6. It shows the regression curve in logarithm expression based on different delay time (P  <  0.05). All the possible lagging weeks were simulated for these 6 sites, and then the delay duration with lowest P value (given by SPSS) was chosen for each site.

Results demonstrated that mcyA concentration was the most significant input (with the least P-value) for predicting MC concentration (Table 1). An optimum regression relationship was found between mcyA and MC concentrations in each site when considering site-specific delay period (Table 1, Fig. 3). The MC concentrations at sites 1, 2, 5, and 6 strongly correlated with mcyA concentration obtained two weeks beforehand (P < 0.05). This result indicated that MCs were released into the surrounding water two weeks after the growth of toxigenic *Microcystis*. At sites 3 and 4, MC concentration strongly correlated with mcyA concentration obtained a week ahead of time (P < 0.05). This result demonstrated that the delay period of MC release was only one week at these two sites.

Since the underlying quantitative toxin release characteristics of MCs were identified, the models enabled the prediction of MCs one or two weeks in advance, when real-time qPCR tests were used on the basis of continuous weekly sampling. This prediction method supports a new framework for the risk management of water bodies with cyanobacterial bloom and MC pollution. Toxigenic *Microcystis* abundance can be used as a significant indicator for the early warning of potential MC hazards, with the corresponding qPCR test widely applied in the lake monitoring.

### Different MC release delays and nutrient conditions

No relation was found between toxigenic *Microcystis* abundance and different toxin release delay periods in the Yangcheng Lake. The delay of MC release possibly depends on other factors. The difference in time delays in the models is discussed below, with the focus on nutrient conditions.

In the Yangcheng Lake, Wilcoxon signed ranks tests were used to cross-verify differences in environmental factors that include TN, TP, NH_3_-N, TN:TP ratio, and NH_3_-N:TN ratio between sites with different toxin release delays. Sites with different *τ* values significantly differed (P < 0.1) in NH_3_-N:TN and TN:TP ratios (Table 2). This result validates the previous finding that nutrients affect MC release. In addition, for the eutrophic Yangcheng Lake, nutrient ratios rather than individual nutrient concentration level influence the delay period.

In the Yangcheng Lake, statistical test results suggested that both NH_3_-N:TN and TN:TP ratios may cause the MC release delay to vary between one and two weeks. On the basis of the differences in delay period and the characteristics of nutrient ratios, the six sites were classified into three groups: sites 1, 2, and 5 in group one; sites 3 and 4 in group two; and site 6 in group three. Group 1 showed significantly higher NH_3_-N:TN level and lower TN:TP level than Group 2, whereas Group 3 had significantly lower NH_3_-N:TN and higher TN:TP ratios than Group 2. Descriptive statistics of nutrient ratios for three groups are shown in Table 3. This result suggests that the delay period is shortened to one week when the NH_3_-N:TN and TN:TP ratios are within a certain range. Taking the medians of nutrient ratios (0.015 for NH_3_-N:TN and 9.2 for TN:TP) in group 2 as references, we hypothesized that NH_3_-N:TN and TN:TP ratios of approximately 0.015 and 9.2 possibly shortened the delay in MC release. Further research should explore the quantitative mechanisms underlying the time scale of MC release delay and potential influential factors.

### Potential model applications in MC risk management

The data obtained from weekly monitoring of MC concentration in the Yangcheng Lake in 2012 did not exceed the WHO recommended value of 1.0 μg/L for dissolved toxins in drinking water. However, MCs considerably accumulated in aquatic products, such as fish and crabs, and fish consumption was considered as a main channel for human exposure^30^,^31^,^32^. The highest estimated daily intake for bioaccumulation is 38 times higher than the WHO recommendable maximum daily intake^33^,^34^. The associated toxic effect posed by MCs on water and food from the lake, as a large-scale source for potable water and freshwater products, is bound to be a concern. Potential applications of the prediction model in MC risk management are discussed below.

The model is helpful to formulate site-specific monitoring plans on toxigenic *Microcystis* detection. The quantitative relationship between MCs and toxigenic *Microcystis* showed a variation in the slopes of the regression curves, with the largest slope at site 2 (0.094) and the smallest slope at site 1 (0.034). The slopes indicate the sensitivity of MC concentration to the change in the amount of toxigenic *Microcystis*. The MC concentration at sites with steep slopes (e.g., site 2) would sharply change even with a minimal variation in mcyA concentration. As a result, a high sampling frequency is needed for mcyA measurements until mcyA reaches its peak value to predict an unexpected augmentation of MC risk. Furthermore, the monitoring frequency of mcyA value must be increased at sites with short delay periods. For example, the MC concentration at site 3 changes with mcyA after a week of delay. Thus, monitoring should be performed more frequently than every week from late August to early October. By contrast, at site 1, the delay period for MC release is two weeks and the MC concentration would not change significantly when mcyA concentration varies. Therefore, mcyA content could be measured once every two weeks to predict MC risk. Consequently, a rough guidance on mcyA test frequency for the Yangcheng Lake aiming at MC risk warning was proposed (Table 4).

The model can also be used to derive different warning levels of mcyA concentration in accordance with MC control requirements. To make the process convenient and time efficient, regression curves of various delay periods for the six sites were integrated into *Y*_*t*_* = 0.056ln[X*_*(t−τ)*_] − *0.407* (*τ* = 1 or 2; P < 0.001) for the entire lake. Once mcyA is measured at a specific location, for the western and eastern areas of the Yangcheng Lake, the concentration of MC two weeks later can be estimated using the above equation. The concentration of MC one week later in the inner area of the Yangcheng Lake can also be estimated using the equation. The confidence interval for MC concentration was narrowed down compared with the separate equations for individual sites. The P-value also confirmed an apparent uncertainty decrease (P < 0.001). This generalized equation can be used to infer the threshold of toxigenic *Microcystis* abundance once the MC concentration limit is pre-set. For instance, a two-level warning threshold was obtained by the inverse calculation when the extracellular MC control limit was set to 1.0 μg/L (standard compliance value for drinking water source) and 0.5 μg/L (assuming daily MC exposure from drinking water is approximately equal to the exposure from fish consumption^31^). The calculation results suggested that the lower and higher warning level of mcyA concentration should be set as 1.0 × 10^7^ and 8.0 × 10^10^ copies/L, whose corresponding 95% confidence intervals of predicted MC concentration are [0.45, 0.55] and [0.79, 1.20] μg/L, respectively. Taking the model uncertainty into account, the threshold value can be lowered or raised for the sake of more conservative or more adventurous risk management. For instance, the threshold 1.0 × 10^7^ copies/L could be lowered down to 7.2 × 10^6^ copies/L or raised up to 2.3 × 10^7^ copies/L, while the probability that MC concentration exceeds 0.5 μg/L would be reduced from 50% to 10% or increased from 50% to 90%.

## Methods

### Sample collection and processing

The Yangcheng Lake, located between the Taihu Lake and the Yangtze River in China, covers an area of 120 km^2^. This lake is a drinking water source for Kunshan City and a backup water source for Suzhou City, serving a population of 11 million. Moreover, the lake is commercially important because of fishery production. The Yangcheng Lake has been repeatedly subjected to toxic algal blooms and microcystin pollution since the 1990 s because of wastewater inflow and aquaculture development. Monitoring and controlling the MCs in the Yangcheng Lake is a government priority in consideration of human health protection and economic growth in the region.

A total of 81 water samples from six sites of the Yangcheng Lake were collected weekly from July to October in 2012. The sampling sites covered the western, middle, and eastern sections of the lake (sites 1 to 6). The corresponding weeks were numbered as 1 to 3 (July), 4 to 7 (August), 8 to 12 (September), and 13 to 15 (October).

Routine water quality parameters, including water temperature, pH, dissolved oxygen concentration, Chla concentration, and cyanobacterial biomass, were measured on site with a multi-probe sensor (YSI 6600, USA). Approximately 1 L of water was collected with a vertical water sampler at 0.5 m depth under the water surface, instantly stored on ice, and then delivered to the laboratory for further analysis. Nutrients including NH_3_-N, TN, and TP were analyzed in accordance with standard protocols^35^.

### Real-time qPCR for total and toxigenic *Microcystis* quantification

Water samples (100 mL) were centrifuged at 12000 rpm for 10 min at 4 °C as previously described, and DNA was extracted using Universal Genomic DNA Extraction Kit Ver.3.0 in accordance with the manufacturer’s instruction (TaKaRa Code: DV811A). For each sample, 50 μL of DNA was obtained and stored at −80 °C for preparation.

The total and toxigenic *Microcystis* were quantified on the basis of the copy numbers of the 16S rDNA and mcyA genes, respectively^36^,^37^,^38^. Amplification was performed using 16S rDNA primers (16S-F: 5-TATTGGGCGTAAAGCGTCCT-3; 16S-R: 5-AACCACATACTCCACCGCTT-3) and mcyA primers (mcyA-F: 5-TTTCATCTCCATCGCCGCA-3; mcyA-R: 5-GGACTCCCACTTGTTTACCGA-3). The total and toxigenic *Microcystis* sequences were 396 and 376 bp long, respectively. Real-time qPCR was conducted using an Applied Biosystems 7500 Fast Real-Time PCR System (USA). Subsequently, 1 μL of DNA template, 0.1 μL of (20 μM) primers, 5 μL of 2 × SYBR Premix Ex Taq^TM^ (TaKaRa, Dalian, China), and 3.8 μL of dH_2_O were mixed. PCR parameters include 95 °C for 10 s; 40 cycles of 95 °C for 5 s, 59 °C for 30 s, and 72 °C for 30 s; melting curve analysis at 95 °C for 15 s; and annealing at 60 °C for 1 min. Standard curves were prepared on the basis of diluted plasmid DNA, and double distilled water was used as the negative control. Purified plasmid cDNA was quantified using UV spectrophotometry (260 nm). The copy numbers of 16S rDNA and mcyA were determined through qPCR to represent the relative abundance of total and toxigenic *Microcystis*, respectively^39^.

In our previous study, the relationships between the cell numbers of *M. aeruginosa* counted by microscopy and the copy numbers of the 16S rDNA and mcyA genes detected by qPCR indicated highly significant correlation coefficients. Linear regression relationships of 16S rDNA (y = 0.989x, R^2^ = 0.8087) and mcyA (y = 1.102x, R^2^ = 0.8863) to cell numbers were then used to estimate the cell number of *Microcystis* and potentially toxigenic *Microcystis* genotypes in each sample. These linear relationships suggest that *M. aeruginosa* (FACHB-905) contains one copy number each of 16S rDNA and mcyA genes. This suggestion is consistent with previous studies that showed equivalent cells of total and toxigenic *Microcystis* calculated from the copy number of the 16S rDNA^40^ and mcyD genes^41^. However, the number of gene copies in each *Microcystis* cell may vary. Kaneko *et al.*^42^ showed that two copies of the mcy gene are carried by each *Microcystis* cell and that 4 to 250 copies of the 16S rDNA gene are carried per *Microcystis* cell^40^,^43^. In the present study, the copy numbers of the 16S rDNA and mcyA genes were used to indicate the abundances of total and toxigenic *Microcystis* in water samples.

### Detection of MCs

Enzyme-linked immunosorbent assay kits (J&Q Environmental Technologies Co., Ltd.) were used to measure MC concentration in triplicate^44^. MC-monoclonal antibody was used, which can react with a majority of the microcystins-producing variants, thereby representing the total MCs^45^. The concentration of the complete antigen was 5 μg/mL. The dilution of the monoclonal antibody and enzyme tracer was 1:3000. The detection limit was 0.05 μg/L.

### Statistical analysis

All data were processed and analyzed using SPSS Statistics 18.0 (USA). The quantitative relationship between toxigenic *Microcystis* and extracellular MCs was deduced from a nonlinear regression process (P < 0.05). Wilcoxon signed-ranks tests on the NH_3_-N:TN and TN:TP ratios between the sites were employed to identify possible effects of these nutrient levels on the differences in the delay period for MC release. Data obtained at the same time from two sites were paired and compared.

## Additional Information

**How to cite this article**: Dong, X. *et al.* Extracellular microcystin prediction based on toxigenic *Microcystis* detection in a eutrophic lake. *Sci. Rep.* **6**, 20886; doi: 10.1038/srep20886 (2016).

## Figures and Tables

**Table 1:** P values of regression models using different input variables (Chla, cyanobacteria, 16S rDNA, and mcyA) with different delaying time for six sites.

P value of regression model as *Y*_*t*_ = *αX*_*t−τ *_*+ β*		*τ* = 0 week	*τ* = 1 week	*τ* = 2 weeks	*τ* = 3 weeks	*τ* = 4 weeks
Site 1	X: Chla	0.159	0.689	0.050	0.209	0.204
	X: *ln*(cyanobacteria)	0.074	0.550	0.130	0.267	0.359
	X: *ln*(16s rDNA)	0.006	0.685	0.308	0.662	0.237
	X: *ln*(mcyA)	0.092	0.232	**0.030***α* = 0.035*β* = −0.139	0.170	0.314
Site 2	X: Chla	0.597	0.847	0.251	0.601	0.673
	X: *ln*(cyanobacteria)	0.138	0.465	0.926	0.411	0.010
	X: *ln*(16s rDNA)	0.386	0.606	0.048	0.283	0.720
	X: *ln*(mcyA)	0.622	0.037	**0.008***α* = 0.093*β* = −0.852	0.036	0.294
Site 3	X: Chla	0.139	0.809	0.142	0.494	0.055
	X: *ln*(cyanobacteria)	0.283	0.836	0.798	0.171	0.735
	X: *ln*(16s rDNA)	0.518	0.627	0.415	0.046	0.208
	X: *ln*(mcyA)	0.093	**0.028***α* = 0.059*β* = −0.475	0.065	0.108	0.830
Site 4	X: Chla	0.040	0.438	0.078	0.794	0.307
	X: *ln*(cyanobacteria)	0.688	0.443	0.158	0.493	0.714
	X: *ln*(16s rDNA)	0.180	0.492	0.189	0.903	0.702
	X: *ln*(mcyA)	0.170	**0.039***α* = 0.037*β* = −0.135	0.427	0.723	0.466
Site 5	X: Chla	0.343	0.546	0.171	0.387	0.740
	X: *ln*(cyanobacteria)	0.381	0.319	0.132	0.103	0.289
	X: *ln*(16s rDNA)	0.393	0.461	0.204	0.811	0.050
	X: *ln*(mcyA)	0.373	0.099	**0.005***α* = 0.071*β* = −0.554	0.124	0.037
Site 6	X: Chla	0.897	0.111	0.039	0.094	0.638
	X: *ln*(cyanobacteria)	0.082	0.150	1.000	0.690	0.635
	X: *ln*(16s rDNA)	0.601	0.190	0.061	0.010	0.142
	X: *ln*(mcyA)	0.295	0.051	**0.004***α* = 0.072*β* = −0.732	0.022	0.079

**Table 2:** Wilcoxon signed ranks test results of NH_3_-N:TN and TN:TP ratio between every two sites.

	Test on difference between NH_3_-N:TN		Test on difference between TN:TP	
	Site 3**	Site 4**	Site 3**	Site 4**
Site 1*	lower than site 1 (P: 0.011)	lower than site 1 (P: 0.075)	higher than site 1 (P: 0.005)	higher than site 1 (P: 0.047)
Site 2*	lower than site 2 (P: 0.004)	no significant difference	higher than site 2 (P: 0.017)	no significant difference
Site 5*	lower than site 5 (P: 0.033)	lower than site 5 (P: 0.023)	higher than site 5 (P: 0.050)	higher than site 5 (P: 0.005)
Site 6*	higher than site 6 (P: 0.007)	higher than site 6 (P: 0.003)	no significant difference	lower than site 6 (P: 0.007)

^*^: site with two-week delay of MC release; **: site with one-week delay of MC release.

**Table 3:** Descriptive statistics of nutrient ratios at different sites.

Nutrient ratio	NH_3_-N:TN			TN:TP		
	Mean	Median	Standard Deviation	Mean	Median	Standard Deviation
Group 1 (Site 1, 2, 5)	0.050	0.032	0.064	6.1	5.2	2.7
Group 2 (Site 3, 4)	0.029	0.015	0.059	10.0	9.2	3.3
Group 3 (Site 6)	0.007	0.006	0.003	13.8	13.9	2.8

**Table 4:** Suggestion on mcyA test frequency at six sites considering the magnitudes of model slope and parameter *τ.*

Test period	Site 1	Site 2, 3, and 5	Site 4 and 6
From August to October	Once every two weeks	Once every week	Once every two weeks ~ Once every week
Other months	Once every month or even lower frequency at all sites		
